# Supplementary material for: Resolving the full spectrum of human genome variation using Linked-Reads
Source: Genome Res. 2019 Apr;29(4):635–45. doi: 10.1101/gr.234443.118 (PMC6442396; doi:10.1101/gr.234443.118)
Supplement: Supplemental Material [file supp_gr.234443.118_Supplemental_longranger-2.2.zip › longranger-2.2.tar.bz2!/lr2.2-public/lib/stan/cmdstan/stan/doc/api/html/agrad_2rev_2functions_2log1p__exp_8hpp_source.html]

Stan: src/stan/agrad/rev/functions/log1p\_exp.hpp Source File


|  |  |
| --- | --- |
|  | Stan  2.5.0  probability, sampling & optimization |


- Main Page
- Related Pages
- Namespaces
- Classes
- Files

- File List
- File Members

All Classes Namespaces Files Functions Variables Typedefs Enumerations Enumerator Friends Macros Pages

- src
- stan
- agrad
- rev
- functions

log1p\_exp.hpp

Go to the documentation of this file.

1 #ifndef STAN\_\_AGRAD\_\_REV\_\_FUNCTIONS\_\_LOG1P\_EXP\_HPP

2 #define STAN\_\_AGRAD\_\_REV\_\_FUNCTIONS\_\_LOG1P\_EXP\_HPP

3

4 #include <stan/agrad/rev/var.hpp>

5 #include <stan/agrad/rev/internal/v\_vari.hpp>

6 #include <stan/math/functions/log1p\_exp.hpp>

7 #include <stan/agrad/rev/calculate\_chain.hpp>

8

9 namespace stan {

10  namespace agrad {

11

12  namespace {

13  class log1p\_exp\_v\_vari : public op\_v\_vari {

14  public:

15  log1p\_exp\_v\_vari(vari\* avi) :

16  op\_v\_vari(stan::math::log1p\_exp(avi->val\_),

17  avi) {

18  }

19  void chain() {

20  avi\_->adj\_ += adj\_ \* calculate\_chain(avi\_->val\_, val\_);

21  }

22  };

23  }

24

29  inline var log1p\_exp(const stan::agrad::var& a) {

30  return var(new log1p\_exp\_v\_vari(a.vi\_));

31  }

32

33  }

34 }

35 #endif

stan::agrad::log1p\_exp

fvar< T > log1p\_exp(const fvar< T > &x)

**Definition:** log1p\_exp.hpp:15

log1p\_exp.hpp

stan::agrad::var::vi\_

vari \* vi\_

Pointer to the implementation of this variable.

**Definition:** var.hpp:40

v\_vari.hpp

var.hpp

stan::agrad::calculate\_chain

double calculate\_chain(const double &x, const double &val)

**Definition:** calculate\_chain.hpp:8

stan::agrad::var

Independent (input) and dependent (output) variables for gradients.

**Definition:** var.hpp:27

calculate\_chain.hpp


---

[ Stan Home Page ]

*© 2011–2014,
Stan Development Team.*
